# The effect of hypomagnetic field on survival and mitochondrial functionality of active *Paramacrobiotus experimentalis* females and males of different age

**DOI:** 10.3389/fphys.2023.1253483

**Published:** 2023-09-08

**Authors:** Amit Kumar Nagwani, Anna Budka, Agnieszka Łacka, Łukasz Kaczmarek, Hanna Kmita

**Affiliations:** ^1^ Department of Bioenergetics, Faculty of Biology, Institute of Molecular Biology and Biotechnology, Adam Mickiewicz University in Poznań, Poznań, Poland; ^2^ Department of Mathematical and Statistical Methods, Poznań University of Life Sciences, Poznań, Poland; ^3^ Department of Animal Taxonomy and Ecology, Faculty of Biology, Institute of Environmental Biology, Adam Mickiewicz University in Poznań, Poznań, Poland

**Keywords:** tardigrades, *Paramacrobiotus experimentalis*, hypomagnetic field, survival, the mitochondrial inner membrane potential (Δψ), age, sex

## Abstract

Even for tardigrades, often called the toughest animals on Earth, a hypomagnetic field (HMF) is an extreme environment. However, studies on the effect of HMF on tardigrades and other invertebrates are scarce. Mitochondria play an important role in an organism’s response to extreme conditions. The effect of HMF on the mitochondrial inner membrane potential (Δψ), a well-known marker of mitochondria functionality, shows that mitochondria are very sensitive to HMF. To measure the HMF effect on *Paramacrobiotus experimentalis*, we calculated the tardigrade survival rate and Δψ level after HMF treatments of different durations. We also estimated the relationship between the age and sex of the tardigrade and the HMF effect. We observed age- and sex-related differences in Δψ and found that Δψ changes after HMF treatment were dependent on its duration as well as the animal’s age and sex. Furthermore, active *P. experimentalis* individuals displayed a high survival rate after HMF treatment. The data may contribute to the understanding of tardigrade aging and their resistance to extreme conditions including HMF, which in turn may be useful for future space explorations.

## Introduction

The Earth’s magnetic field, also called the geomagnetic field (∼50 μT) or standard magnetic field (SMF), is regarded as a crucial factor for living organisms because it protects organisms living on Earth from corpuscular radiation, e.g., solar wind and cosmic radiation (for review, see, e.g., Xue et al., 2021; Erdmann et al., 2021a). However, it decreases significantly with increasing distance from the surface of the Earth and can be blocked or shielded on Earth, resulting in a hypomagnetic field (HMF; <5 μT) (e.g., Mo et al., 2014). Thus, exposure to HMF may happen not only during space travel but also on Earth, e.g., in buildings with steel walls or steel reinforcements (Sinčák and Sedlakova-Kadukova, 2023). The exposure is known to affect living organisms at different biological levels (Conley, 1970; Binhi and Prato, 2017; Erdmann et al., 2021a; Sinčák and Sedlakova-Kadukova, 2023). Therefore, HMF can be regarded as an extreme condition but the mechanisms underlying HMF’s effect on living organisms are still largely unclear (e.g., Sinčák and Sedlakova-Kadukova, 2023). At a cellular level, mitochondria have been suggested as the organelle most sensitive to HMF (e.g., Fu et al., 2016; Zhang and Tian, 2020). The mitochondrial inner membrane potential (Δψ) is an essential component for healthy mitochondrial functioning, and mitochondrial dysfunction co-occurs with Δψ decrease (Cao et al., 2004; Srinivasan et al., 2017). Thus, the decrease in Δψ is considered to be an indicator of reduced cell health (Wang et al., 2019). Importantly, a decrease in Δψ has been reported under extreme conditions (e.g., Neginskaya et al., 2021), including exposure to HMF (Fu et al., 2016; Wang et al., 2022).

Studies on the effect of HMF on humans and other vertebrates as well as plants are quite common, whereas the studies on invertebrates are still limited, particularly for those displaying cryptobiosis capability (e.g., Erdmann et al., 2017; Erdmann et al., 2021b; Sinčák and Sedlakova-Kadukova, 2023). Tardigrades are microinvertebrates (mean size of ca. 500 μm) which inhabit almost all terrestrial and aquatic ecosystems throughout the world (Nelson et al., 2015). These animals are commonly known as the toughest animals on Earth, (Copley, 1999), due to their resistance to different environmental stressors. These include lack of water, low and high temperatures, high doses of radiation, low and high atmospheric pressure, low gravity, and high concentration of different toxins (e.g., Crowe, 1975; Ramlov and Westh, 2001; Altiero et al., 2011; Guidetti et al., 2012; Kaczmarek et al., 2019; Hesgrove and Boothby, 2020). Thus, tardigrades are excellent models for research on mechanisms of adaptation to the most extreme environments, including exposure to space conditions (e.g., Jönsson, 2007; 2020; Guidetti et al., 2012; Horikawa, 2012; Schill and Hengherr, 2018; Rebecchi et al., 2020; Arakawa, 2022), particularly as results obtained from studies on tardigrades can be extrapolated and applied to vertebrates, including humans (Hashimoto et al., 2016).

Up to now, ca. 1,500 tardigrade species have been described (Degma et al., 2023) but only three species have been used in studies on mortality in the presence of HMF (Erdmann et al., 2017; 2021b). They are *Hypsibius exemplaris* (misidentified in earlier works as *H. dujardini*) (Gąsiorek et al., 2018), *Echiniscus testudo* (Doyère, 1840), and *Milnesium inceptum* (Morek et al., 2019), all represented by parthenogenetic females and studied during three stages of anhydrobiosis (Erdmann et al., 2017; Erdmann et al., 2021b). The authors of these studies concluded that HMF significantly increased the mortality of the studied species and decreased their capability of anhydrobiosis, probably due to impairment of metabolic processes, although there were differences in HMF tolerance between the studied species. This aligns with the available data on tardigrade differentiation of tolerance to extreme factors as has been reviewed in relation to anhydrobiosis, (Nagwani et al., 2022), as well as the reported observation that mitochondrial functionality is a prerequisite for anhydrobiosis survival (Halberg et al., 2013; Wojciechowska et al., 2021a). Accordingly, it is well known that mitochondria play a central role in cell metabolism and control of stress responses, and mediate various cellular outcomes (e.g., Vakifahmetoglu-Norberg et al., 2017). However, the impact of HMF on tardigrade mitochondria functioning has not yet been studied.

It has been reported that the length of exposure to HMF may be an important factor for the observed effect (Sinčák and Sedlakova-Kadukova, 2023) Moreover, it appears that the tardigrade’s ability to survive under extreme conditions can be influenced by many factors (e.g., McCarthy and delBarco-Trillo, 2020). Our previous results indicate that age and sex could be important to tardigrades’ survival under extreme conditions represented by water deficiency resulting in dehydration (Nagwani et al., 2023). Therefore, we decided to study the impact of different durations of exposure to HMF on the survival rate and mitochondrial Δψ levels of active tardigrades of different ages and sex. The obtained data provide insight into age- and sex-dependent changes in tardigrade mitochondria functionality that may contribute to animals’ aging, as well as their resistance to HMF.

## Materials and methods

### Reagents

For the detection of the mitochondrial inner membrane potential, tetramethylrhodamine methyl ester (TMRM; ThermoFisher #T668) was applied. Carbonyl cyanide 4-(trifluoromethoxy) phenylhydrazone (FCCP; Sigma-Aldrich #2920) was used to eliminate the mitochondrial inner membrane potential. To stain DNA, 4′,6-diamidino-2-phenylindole (DAPI; Sigma-Aldrich #D9542) was applied. For confocal fluorescence microscopy, specimens were mounted on glass slides (76 mm × 26 mm; bionovo #B-1198) using the Vectashield antifade mounting medium (Vector laboratories #H-1000-10) and glass coverslips (16 mm × 16 mm; ChemLand) #04-298.202.00). For fluorescence quantitative analysis, multi-well plates (Ratiolab #6018717) were applied.

### Cultures of *Paramacrobiotus experimentalis*

Females and males of *P. experimentalis* (Kaczmarek et al., 2020; Nagwani et al., 2023) were reared together in covered, vented plastic Petri dishes (55 mm in diameter), with the bottom scratched with sandpaper to allow the animals to move. The individuals were coated with a thin layer of the culture medium, a mixture of spring water (Żywiec Zdrój S.A., Poland), and ddH2O in a 1:3 ratio. The culture medium was exchanged every week, and animals were fed with the rotifer *Lecane inermis* (Bryce, 1892) (strain 1.A2.15), provided by Dr. Edyta Fiałkowska (Institute of Environmental Sciences, Jagiellonian University, Krakow, Poland). The Petri dishes were kept in the POL EKO KK 115 TOP + climate chamber at 20°C, in the dark, with relative humidity (RH) of 40% (Roszkowska et al., 2021).

Eggs laid by females were collected to obtain different age classes and were cultured as described above. Each of the classes contained females and males, and was kept in separate Petri dishes. The first oviposition took place 19.3 ± 3.6 days after hatching. Females and males were distinguished based on morphological characteristics, e.g., average body length (males are smaller than females), average body width (males are slimmer than females), the presence of eggs in the females’ ovary (Supplementary Table S1) and the body shape. Females have a barrel shape (especially in the late stages of oogenesis) and the posterior part of the body not hooked whereas males do not have the barrel shape but sometimes the posterior part of their body is slightly hooked. The accuracy of the applied approach of identification of sexes was confirmed by detecting of spermatozoa movements in the gonad using a stereomicroscope (OLYMPUS SZ61) and observing gonads using a transmission electron microscope (Hitachi H500). The number of active animals and eggs as well as their body length, body width and body shape were assessed using the stereomicroscope. The calibrated grid of the stereomicroscope was used to measure the body length and body width to an accuracy of 10 µm. Because the average lifespan of the individuals under the laboratory culture conditions is approximately 360 days (our unpublished data), three different age classes were selected, representing the following age ranges: 30–60 days, 150–180 days, and older than 300 days. These age classes were described as growing adults, mature adults, and old adults, respectively (Supplementary Table S1). To determine the average lifespan, 200 eggs were isolated from *P. experimentalis* culture from which 182 were hatched and the hatched individuals were transferred to the culture Petri dishes and cultured as described above. They were observed continuously and their activity was noted until sex distinguishing was possible (see above). Then, 30 females and 30 males were selected and further cultured as described above and observed every 3 days and then every week. The observation was performed until the last female or male died and the number of active individuals at each of the applied time windows of observation was estimated.

### Exposure to a hypomagnetic field

**FIGURE 1:**
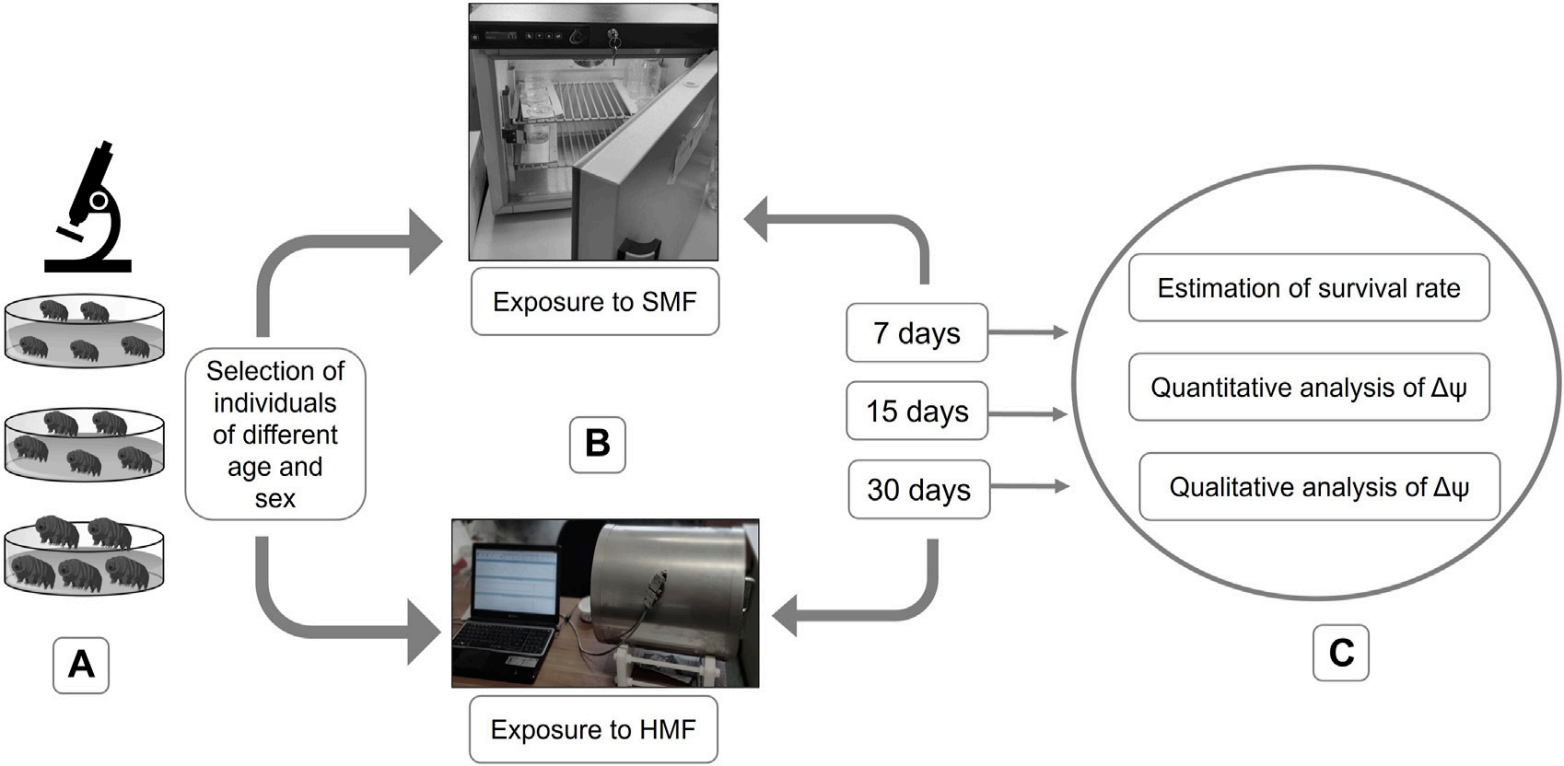
Graphical representation of performed experiments. **(A)** selection of active individuals of different age and sex; **(B)** exposure to SMF or HMF for different durations (i.e., 7, 15, and 30 days); **(C)** post-exposure analysis including estimation of the survival rate and analysis of the level of the mitochondrial inner membrane potential (Δψ).

Females and males of the selected age classes were separated and divided into experimental and control animals. They were placed in covered, vented plastic Petri dishes (55 mm in diameter) with the bottom scratched with sandpaper and coated with a thin layer of the culture medium. The feeding and exchange of culture medium were performed every week. Control females and males were exposed to standard (geomagnetic) field (SMF) using a POL EKO KK 115 TOP + climate chamber whereas experimental females and males were exposed to a hypomagnetic field (HMF) by application of a special anti-magnetic chamber, known as a Chamber Isolated from Magnetic Field (CIMF) (Erdmann et al., 2017). The chamber can deflect the force field by concentrating it inside the material’s substance (Conley, 1970), leading to a 200-fold reduction of SMF (Kopcewicz et al., 1999; Miglierini et al., 2002). Importantly, for both the control and experimental animals, standard culture conditions were applied (temperature of 20°C, darkness (24 h), and RH of 40%). The air movement in the experimental chamber was verified by Erdmann et al. (2017). Importantly, the tardigrade survival rate in their control box and our control chamber during 1 month were not statistically different. The exposure to HMF was performed for 7, 15, and 30 days (Figure 1).

### Estimation of hypomagnetic field effect

The impact of HMF was estimated by calculating the survival rate and evaluating the functional state of mitochondria (Figure 1). To evaluate the effect of HMF on mitochondrial functionality, the level of the mitochondrial inner membrane potential (Δψ), a well-known marker of mitochondria functionality, was estimated.

To calculate the survival rate, groups of 15 active females or males of different ages, each in three repetitions (15 × 3 = 45), were exposed to a given duration of HMF or SMF. After each of the exposure, the culture medium was exchanged and animals were observed at 0, 2, 6, 24, and 48 h following the end of the exposure. The survival rate denotes the number of still-active individuals (i.e., displaying coordinated movements of the body and legs) after a given exposure.

A Nikon A1Rsi confocal microscope connected to a digital camera was used to perform a qualitative analysis of Δψ. For each variant of HMF or SMF treatment, three active females or males from each of the selected age classes were stained for 1 h with 2 μM TMRM (a fluorescent probe that accumulates in the mitochondria, dependent on the level of Δψ) and 1 μg/mL of DAPI (a cell-permeable fluorescent probe that binds to DNA). Next, the stained animals were washed carefully by multiple replacements of the culture medium and subsequently kept for 1 h at room temperature in fresh culture medium to remove any excess probes. Since the applied probes are light-sensitive, all steps of staining and washing were carried out in the dark. After washing, the stained animals were mounted on glass slides using Vectashield antifade mounting medium and glass coverslips. The microscopic observation was performed at a wavelength of fluorescence excitation of 544 nm for TMRM and 359 nm for DAPI. Images were obtained using NIS-Elements Viewer 5.21 software and transformed using ImageJ 2.3.0 software. The NIS-Elements Viewer 5.21 software was provided by the Department of Cellular and Molecular Biology at Adam Mickiewicz University in Poznan, Poland. The ImageJ 2.3.0 software was downloaded from the official website of the National Institutes of Health (https://imagej.nih.gov/ij/download.html).

For quantitative analysis of Δψ, groups of 50-100 active females and males of different ages, each in three repetitions, were exposed to a given duration of HMF or SMF, after which they were stained with 2 μM TMRM for 1 h at room temperature in darkness. Next, the stained animals were washed as described above. After being washed, the stained animals were transferred in 100 μL of the culture medium into multi-well plates. Multiple readings per well were immediately taken using a Tecan Infinite 200 Pro microplate reader at an excitation wavelength of 544 nm and an emission wavelength of 590 nm. The measurements were repeated after incubation of the samples for 45 min with 50 µM FCCP, an uncoupler used as a control in the determination of Δψ level. The data were exported using Tecan i-control software and recalculated to the TMRM Fluorescence Index (FI_TMRM_), representing Δψ sensitivity to FCCP (Wojciechowska et al., 2021b). Because of the differences in body size and the resulting differences in the number of animals in each sample, FI_TMRM_ was recalculated for one animal.

### Statistical analysis

To analyse the effect of HMF or SMF (control) treatment, two-way ANOVA was used. The analysis was performed separately for females and males. In the analysis two dependent variables, i.e., the survival rate corresponding to the number of still-active individuals, and the level of Δψ, reflected by the value of FI_TMRM_, as well as two independent variables, i.e., age (three age classes) and duration of the treatment (7, 15 and 30 days) were applied. Because data concerning the effect of HMF or SMF treatment on the survival rate did not meet conditions necessary for ANOVA, as detected by Fligner-Killeen test, the “aligned rank transformation” approach, available in the ARTool package of R software, was used prior to two-way ANOVA. If significant differences related to the analysed dependent variables were found, Tukey *post hoc* test was performed (*α* = 0.05). Due to limited variability of the survival rate data, correlation analysis between survival and FI_TMRM_ data was not performed. Mean values of active animals and level of Δψ were additionally compared between SMF (control) and HMF treatment as well as between females and males using *t*-test (see Supplementary Tables S2, S3).

## Results

### The effect of HMF on the *Paramacrobiotus experimentalis* survival rate depends on its duration and the animal age and sex

**FIGURE 2:**
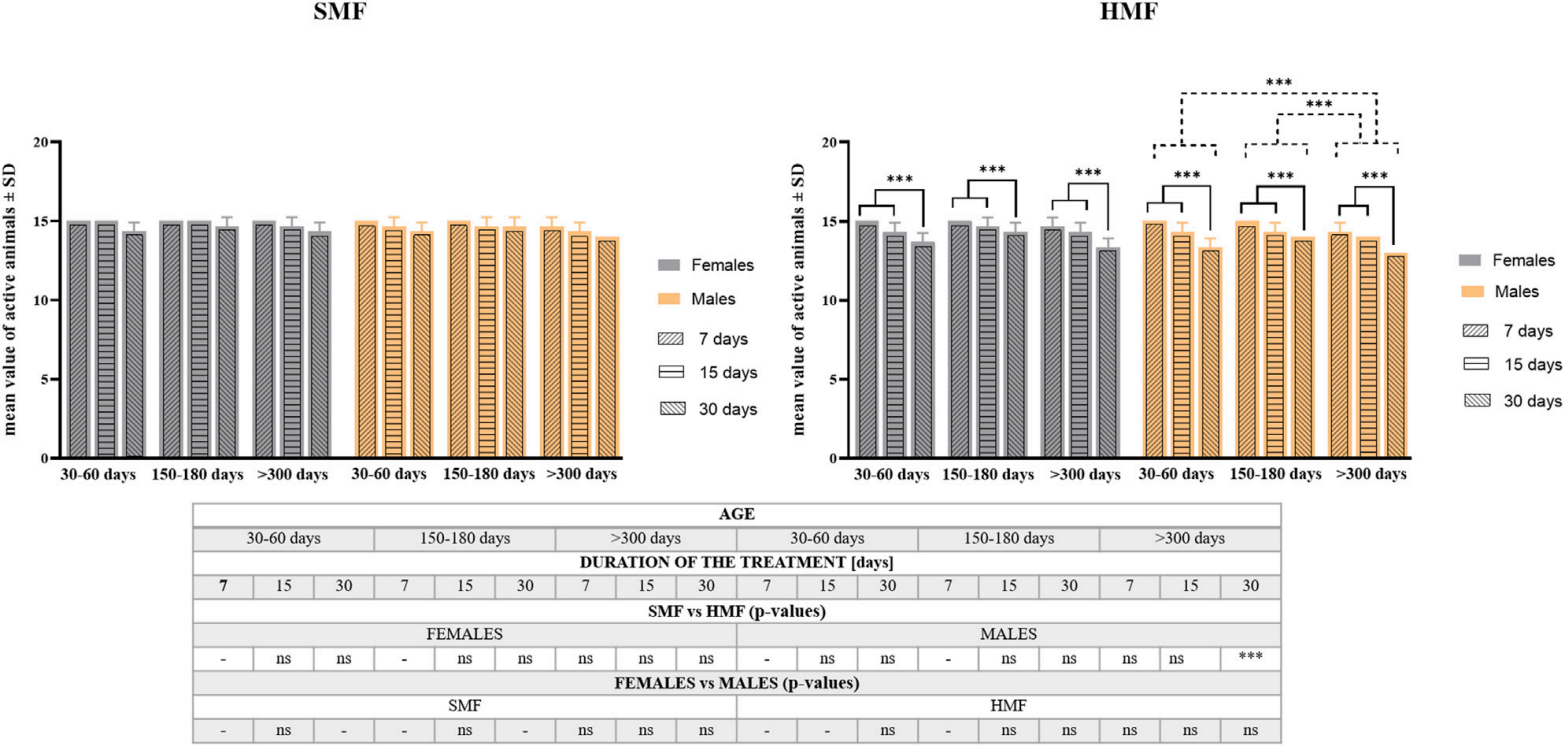
The survival rate of *P. experimentalis* females and males of different age exposed to SMF and HMF of different duration. The survival rate corresponds to mean values ±SD of still active individuals (i.e., displaying coordinated movements of the body and legs) after a given treatment. The age of animals: 30–60 days (growing adults), 150–180 days (mature adults) and >300 days (old adults); durations of the SMF and HMF treatment: 7, 15, and 30 days; statistically significant difference between age classes and treatment durations, dashed and solid lines, respectively. ***p < 0.001. The table presents comparison of the mean values-of active animas between SMF (control) and HMF treatment as well as between females and males using *t*-test. *** and ns denotes *p* < 0.001 and not statistically significant, respectively. When the values for control and experimental groups or for females and males were identical, the analysis was not performed (“-” in the table) (see also Supplementary Table S2).

The effect of exposure to hypomagnetic field (HMF) of different durations (7, 15, and 30 days) on the survival rate was tested for active females and males classified as growing adults (the age of 30–60 days), mature adults (the age of 150–180 days) and old adults (the age of over 300 days) (see also Supplementary Table S1). Animals exposed to the standard magnetic field (SMF) were used as a control. As shown in Figure 2, the age of animals and duration of SMF treatment did not influence the survival rate in a statistically-significant way. However, after HMF treatment, both the duration of exposure and the animal’s age affected the survival rate in a statistically-significant ways that differed between females and males. For females, only the duration of HMF treatment was found to be a statistically significant factor, while for males both the duration of HMF treatment and animal age were statistically significant. For both females and males, 7 and 15-day treatment with HMF resulted in comparable effects on the survival rate that differed significantly from 30-day treatment effect being the strongest one. The survival rate of male growing adults and male mature adults did not differ significantly, but the difference between their survival rate and old adults was significant as the latter were most sensitive to HMF exposure. Nevertheless, survival following HMF treatment by active females and males was high, i.e., no lower after any of the applied treatment variants than 87% (Supplementary Table S2). Moreover, survival of control active females and control active males was not statistically different and the absence of statistically significant difference was also observed between females and males after HMF treatment. When the survival was compared between control animals and animals after HMF treatment, statistically significant difference was only observed for the oldest age class males for the longest HMF duration (Figure 2; Supplementary Table S2). For the longest duration of HMF also initial reduced mobility of females and males was observed but the mobility was restored at the next time point of observation at 2 h following the end of the treatment and did not change until the last time point of observation at 48 h following the end of the treatment.

### The effect of HMF on *Paramacrobiotus experimentalis* mitochondrial Δψ level depends on its duration and the animal’s age and sex

**FIGURE 3:**
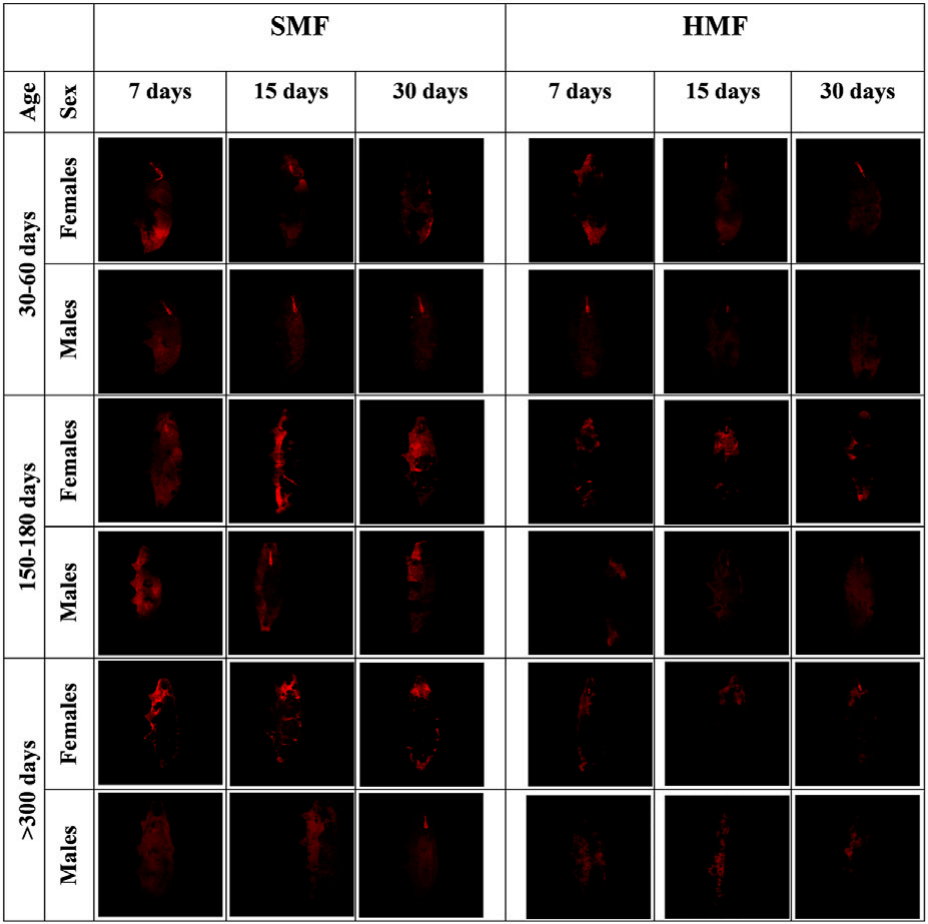
Confocal microscopy images of females and males of different age exposed to SMF and HMF of different duration and stained with TMRM. SMF, standard magnetic field; HMF, hypomagnetic field. The age of the animals: 30–60 days (growing adults), 150–180 days (mature adults), and >300 days (old adults). The duration of SMF or HMF treatment: 7, 15 and 30 days. The figure presents images representative for three animals. All confocal images are maximum projection images taken using the identical settings and later reoriented using ImageJ in order to keep the same orientation of all the captured images.

It is well known that HMF, like other extreme conditions, affects mitochondrial functional state (Fu et al., 2016; Zhang and Tian, 2020). Therefore, we decided to estimate the level of the mitochondrial inner membrane potential (Δψ) for active females and males of different ages and treated with different durations of HMF and SMF, using the intact animals’ TMRM staining and confocal fluorescence microscopy. To assure the specificity of the TMRM staining, the animals were stained with DAPI (Supplementary Figure S1). As shown in Figure 3, the HMF treatment decreased the intensity of the TMRM staining when compared with SMF (control) treatment, suggesting a relevant decrease in Δψ level. The staining intensity also appeared to correlate with the age of active females and males, suggesting age-and sex-dependent changes in Δψ level. The intensity of the TMRM staining appeared to be higher for females and the highest for mature adults and the lowest for growing adults of both sexes, suggesting Δψ level changes related to age. The variation in buccal apparatus visibility could be explained by the amount of ingestion. It is possible that during the time of staining, the ingested food did not reach the gut completely and interfered with the staining and washing steps.

**FIGURE 4:**
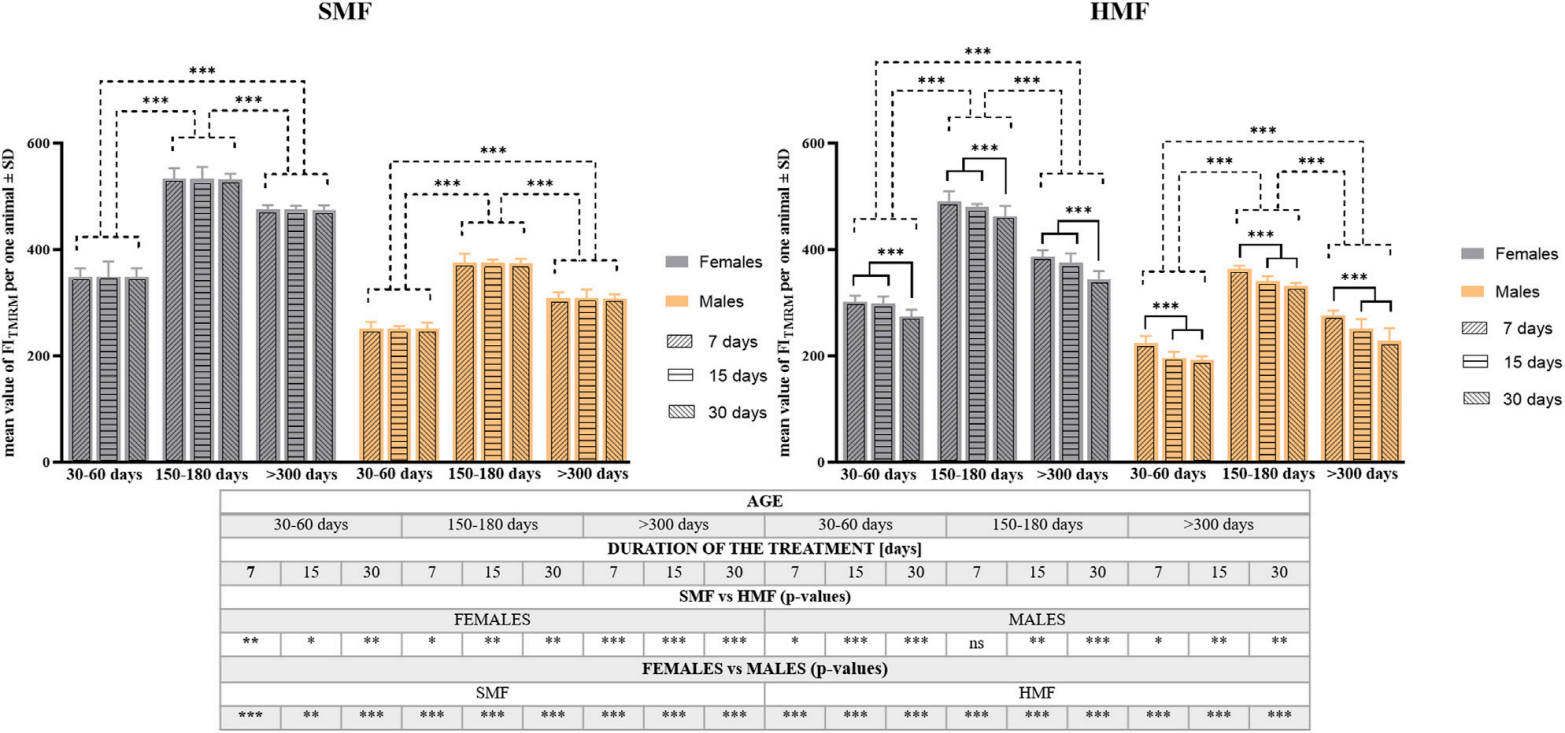
The mitochondrial inner membrane potential (Δψ) level of *P. experimentalis* females and males of different age exposed to SMF and HMF of different duration. The Δψ level corresponds to mean values of FI_TMRM_ ± SD per animal after a given treatment. The age of animals: 30–60 days (growing adults), 150–180 days (mature adults) and >300 days (old adults); durations of the SMF and HMF treatment: 7, 15, and 30 days; statistically significant difference between the age classes and treatment durations, dashed and solid lines, respectively. ***p < 0.001. The table presents results of comparison of Δψ mean values between SMF (control) and HMF treatment as well as between females and males using *t*-test. *, **, ***, ns denotes *p* < 0.05, *p* < 0.01, *p* < 0.001 and not statistically significant, respectively (see also Supplementary Table S3).

To confirm these observations, quantitative analysis of TMRM staining was performed on the animals after HMF and SMF (control) treatment using a microplate reader. The analysis consisted of calculating the TMRM Fluorescence Index (FI_TMRM_) per animal (see also Supplementary Table S3). As shown in Figure 4, relevant values of FI_TMRM_ after SMF and HMF treatments were statistically different (see also Supplementary Table S3), supporting the relevant decrease in Δψ level after HMF treatment. Comparison of FI_TMRM_ between control females and control males as well as between females and males after HMF treatment indicated statistically significant differences for all age classes and treatment duration (see also Supplementary Table S3), that appeared to confirm the higher level of Δψ in females, also after HMF treatment. Moreover, after SMF treatment, the age of animals affected the value of FI_TMRM_ in a statistically-significant way, whereas after HMF treatment both duration and animal age affected the value of FI_TMRM_ in a statistically-significant way. The values of FI_TMRM_ after SMF and HMF treatments were the highest for mature adults and the lowest for growing adults. This accords with the confocal microscopic images and the apparent change of Δψ level related to the animal age. After HMF treatment, the value of FI_TMRM_ was lower for all age classes, indicating the treatment effect on Δψ level. However, for females, 7 and 15-day treatment with HMF resulted in a comparable effect on FI_TMRM_ that differed significantly from the effect of 30-day treatment, while for males significant differences in FI_TMRM_ were observed between 7-day and 15-day treatment as well as between 7-day and 30-day treatment, but not between 15-day and 30-day treatment. This suggested sex-dependent differences in the effect of HMF treatment on Δψ level.

## Discussion

The mitochondrial inner membrane potential (Δψ), applied as a marker of mitochondria functionality, is known to change during aging (e.g., Panel et al., 2018) and under hypomagnetic conditions (e.g., Fu et al., 2016; Zhang and Tian, 2020; Wang et al., 2022; Sinčák and Sedlakova-Kadukova, 2023). However, the age and sex of animals are omitted in HMF studies although their contributions appear to be important for better understanding the mechanisms of resistance to extreme conditions and the process of aging. The newly-described tardigrade species *P. experimentalis* was used in this study, which determined the survival rate of animals and carried out qualitative and quantitative analysis of their Δψ level after SMF (control) and HMF treatment of different durations. The obtained results indicate that tardigrade mitochondria can directly respond to HMF at a functional level by decreasing Δψ levels, and active animals have high survival (determined based on the presence of coordinated movements of the body and legs) of exposure to HMF. On the other hand, the age and sex of the animals are important factors that influence the tardigrade’s response to HMF treatment.

To our knowledge, this is the first report on the survival rates of active tardigrade females and males after exposure to HMF of different durations. The determined survival rate was high, although males, particularly the oldest one, appeared to be more sensitive to HMF. This is reflected by the observation that for females, only the duration of HMF treatment was a statistically-significant factor influencing the survival rate, while for males both the duration of HMF treatment and the animal’s age influenced the survival rate in a statistically-significant way. Moreover, for both females and males, shorter treatments with HMF resulted in comparable effects on the survival rate that differed significantly from the longest treatment effect, while for males significant differences in the survival rate were observed between younger and oldest animals. This accords with existing evidence that, under extreme conditions, females survive better than males (e.g., Zarulli et al., 2018). Although, no statistically significant differences in the survival rate were obtained between females and males after HMF treatment, statistically significant difference was obtained for the oldest age class males for the longest HMF duration when compared with the relevant control SMF variant of the treatment. Thus, the duration of HMF treatment may be an important factor for the observed effect (Sinčák and Sedlakova-Kadukova, 2023), although the male survivability may be affected by their age.

The known data on tardigrade treatment with HMF concern parthenogenetic females of three different species at different stages of anhydrobiosis, i.e., entering anhydrobiosis, during anhydrobiosis, and leaving anhydrobiosis (Erdmann et al., 2017; Erdmann et al., 2021b). In previous experiments (Erdmann et al., 2017; Erdmann et al., 2021b), only one duration of HMF treatment was used, and return to activity was determined. Although some differences were observed for different species, the survival rate was generally low, which was explained by the authors as being due to HMF-mediated impairment of the metabolic processes associated with anhydrobiosis. Accordingly, it has already been established that mitochondrial functionality is indispensable for anhydrobiosis survival (Halberg et al., 2013). Our qualitative and quantitative analysis of *P. experimentalis* Δψ level indicated a relationship between Δψ level and the age of active females and males that makes Δψ level an interesting marker of the tardigrade’s age. The intensity of the TMRM staining was higher for females, which might be an unspecific effect resulting from the females’ bigger body size and the presence of eggs or higher mitochondrial content (e.g., Cardinale et al., 2018), although some data indicate a higher Δψ level in female mitochondria (e.g., Damacena de Angelis et al., 2022).

The level of Δψ differed significantly between SMF (control) and HMF treatment as well as between females and males. However, it was the highest for mature adults and the lowest for growing adults. Besides the age of the animals, the important factor for the effect of HMF treatment was its duration. For females, shorter treatments with HMF resulted in a comparable effect on Δψ level that differed significantly from the effect of the longest treatment. This resembles the effect of HMF treatment duration on the female survival rate. For males, the effects of HMF duration on the survival rate and changes in Δψ level did not overlap; for the former, significant differences were observed between two shorter and the longest durations of HMF treatments, and for the latter, there was only a significant difference between the shortest treatment and the two longer ones. This suggested sex-related differences in sensitivity of Δψ to HMF duration. At present, we do not have an explanation for this, although the observation is in line with known sex-associated differences in mitochondrial function (e.g., Silaidos et al., 2018).

The mechanism by which the level of Δψ is modulated by SMF and HMF remains unclear. Nevertheless, it is known that HMF treatment decreases Δψ and consequently the viability of animal cells, (e.g., Fu et al., 2016; Srimai et al., 2020) which results in a range of adverse effects and severe dysfunctions in animals, including humans (for review, see, e.g., Erdman et al., 2021a). Considering that the magnetic field acts on elements with a magnetic moment, the decrease in Δψ is proposed to be caused by HMF interaction with electrons moving along the mitochondrial respiratory chain, ions moving along ion channels, or proton translocation by the respiratory chain (Binhi and Prato, 2017; Ogneva et al., 2020). When the external magnetic field is changed from SMF to HMF, conditions are no longer optimal for these processes and their efficiency is reduced. It has been also observed that the HMF effect on mitochondria may result in a change to the number of mitochondria and their morphology, which in the case of skeletal muscle diminishes animals’ physical activity (Hu et al., 2020). Importantly, our data indicate that the physical activity of tardigrades was only initially and transiently attenuated after the longest HMF treatment. In addition, reactive oxygen species (ROS) are hypothesized to mediate the effect of HMF on mitochondria (Zhang and Tian, 2020) but available data do not allow for this hypothesis to be validated for tardigrades.

## Conclusion

Our data indicate that Δψ level could be a useful marker of the tardigrade *P. experimentalis* age. Moreover, the tardigrade sensitivity to HMF treatment including Δψ changes may also serve as the marker. Using an accurate marker of tardigrade age may contribute to an explanation of tardigrade aging. The obtained results also provide important data concerning age- and sex-related changes in mitochondrial functioning under extreme conditions. Similar to other animals, *P. experimentalis* responds to HMF treatment with Δψ decrease, but the decrease does not result in impaired survival as reflected by the animal’s physical activity although the activity was shortly and transiently attenuated after the longest HMF treatment. Thus, the long-term effects of the Δψ decrease on the tardigrade longevity and/or reproduction should be further studied to fully explain biological significance of HMF treatment. The observed relationship between the decrease in Δψ level and duration of HMF treatment suggests that HMF effect on mitochondria functionality increases over time. This observation is useful for creating models relevant to long-term space missions, where continuous exposure to HMF is a potential risk factor for different organisms, including humans. Nevertheless, the studied factors should also be considered in studies of HMF effect on other tardigrades species.

## Data Availability

The original contributions presented in the study are included in the article/Supplementary Material, further inquiries can be directed to the corresponding author.
