# Supplementary material for: The effect of hypomagnetic field on survival and mitochondrial functionality of active Paramacrobiotus experimentalis females and males of different age
Source: Front Physiol. 2023 Sep 8;14:1253483. doi: 10.3389/fphys.2023.1253483 (PMC10514487; doi:10.3389/fphys.2023.1253483)
Supplement: Supplementary file 3 [file Table1.DOCX]

**Supplementary Table 1:** The applied age classes distinguished for *P. experimentalis* and based on the selected life history trait analysis. Body length, body width and numbers of eggs were assessed using a stereomicroscope (OLYMPUS SZ61). The calibrated grid of the stereomicroscope was used to measure the body length and body width to an accuracy of 10 µm. ND, no data recorded; n, the number of individuals.

| Age classes | Age range in days  (assigned group name) | Average body length  (± SD) [μm] | | Average body width  (± SD) [μm] | | Average number of  laid eggs per female  (± SD) |
| --- | --- | --- | --- | --- | --- | --- |
|  |  | **Females** | **Males** | **Females** | **Males** |  |
| 1 | 30-60 days  (growing adults) | 420 ± 20  n=25 | 350 ± 30  n=25 | 160 ± 20  n=25 | 110 ± 10  n=25 | 97 ± 18  n=100 |
| 2 | 150-180 days  (mature adults) | 750 ± 40  n=20 | 590 ± 50  n=20 | 240 ± 30  n=20 | 140 ± 20  n=20 | 162 ± 32  n=70 |
| 3 | >300 days  (old adults) | 750 ± 30  n=10 | 600 ± 30  n=10 | 250 ± 30  n=10 | 150 ± 20  n=10 | ND |
