# Supplementary figures and images for: The effect of hypomagnetic field on survival and mitochondrial functionality of active Paramacrobiotus experimentalis females and males of different age
Source: Front Physiol. 2023 Sep 8;14:1253483. doi: 10.3389/fphys.2023.1253483 (PMC10514487; doi:10.3389/fphys.2023.1253483)

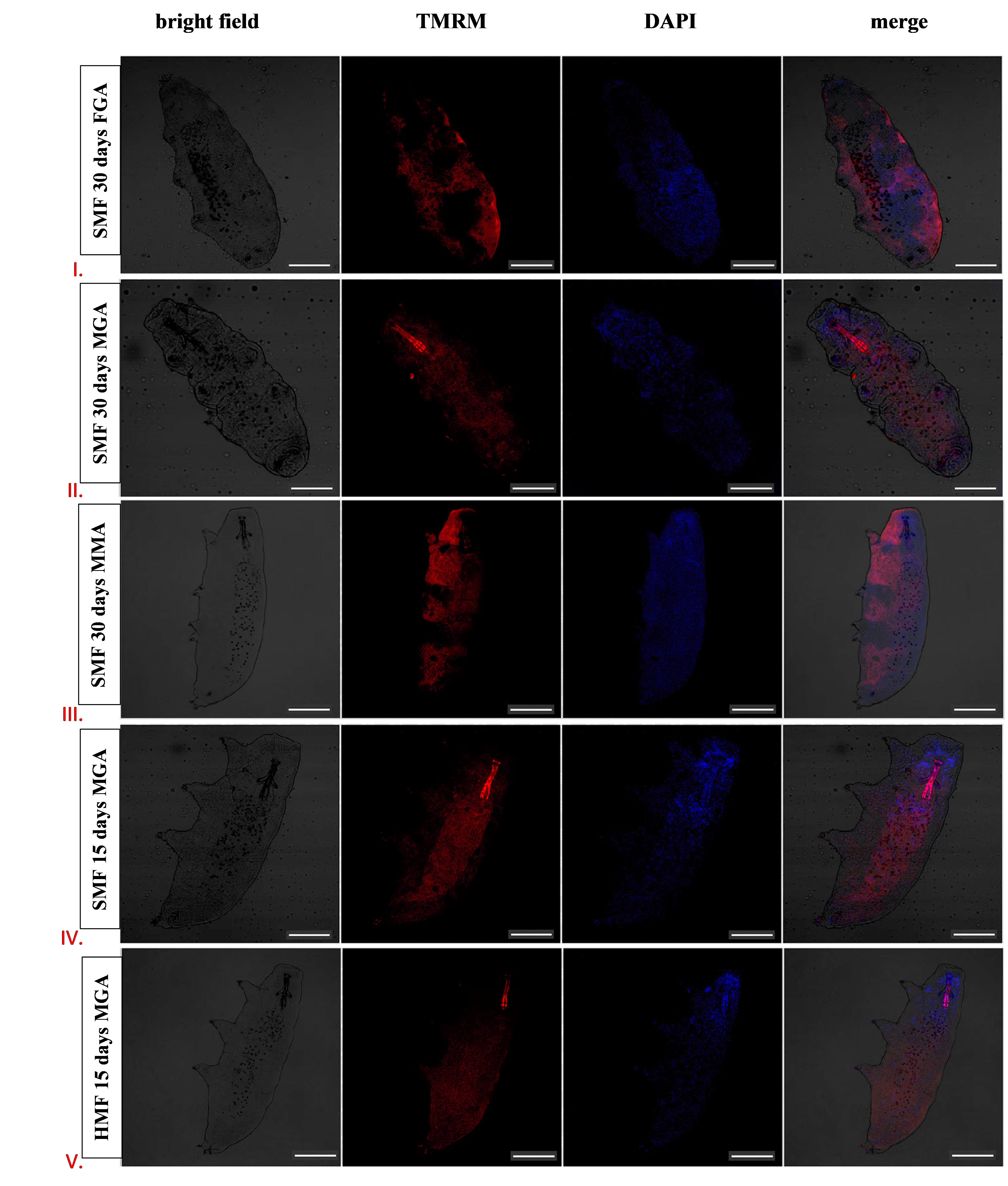

Supplement: Supplementary file 4 [file Image1.TIF]
